# 

**Published:** 1892-01-16

**Authors:** 


BOVRIL For INFLUENZA.—BOVRIL for the last two years has established itself throughout Europe and America as the most perfect antidote, the most certain preventive and approved cure of the [illegible]demic in all its stages. BOVRIL (the product of prime ox [illegible]f) furnishes to the system that stamina and vitality which can [illegible]st counteract the influence of the scourge, and carries the feeblest [illegible]valid through the climax of the attack to a rapid recovery.

A MAGISTRATE'S OPINION.—To the Editor of the "Daily Post."—Sir,—On Thursday I had a smart touch of Influenza, and have achieved such a rapid cure that it may be of service to your readers to know the means used. On Thursday I was totally incapacitated, on Friday convalescent, on Saturday quite well. Treatment each day—four dessert spoonfuls Cod-Liver Oil, one after each meal; four Teacups full of BOVRIL mid-way between meals, and last thing at night. I believe this will cure any one.—Yours, &c., (Signed) George S. Hazlehurst, The Grange, Rock Ferry. J.P., Flintshire.

